# Biosynthesis of Polyhydroxyalkanoate Terpolymer from Methanol via the Reverse β-Oxidation Pathway in the Presence of Lanthanide

**DOI:** 10.3390/microorganisms10010184

**Published:** 2022-01-15

**Authors:** Izumi Orita, Gento Unno, Risa Kato, Toshiaki Fukui

**Affiliations:** School of Life Science and Technology, Tokyo Institute of Technology, 4259 Nagatsuta, Midori-ku, Yokohama 226-8501, Japan; orita.i.aa@m.titech.ac.jp (I.O.); gento910@gmail.com (G.U.); kato.r.ac@m.titech.ac.jp (R.K.)

**Keywords:** polyhydroxyalkanoates, methanol, methylotroph, lanthanide, methylotrophy

## Abstract

*Methylorubrum extorquens* AM1 is the attractive platform for the production of value-added products from methanol. We previously demonstrated that *M. extorquens* equipped with PHA synthase with broad substrate specificity synthesized polyhydroxyalkanoates (PHAs) composed of (*R*)-3-hydroxybutyrate and small fraction of (*R*)-3-hydroxyvalerate (3HV) and (*R*)-3-hydroxyhexanoate (3HHx) units on methanol. This study further engineered *M. extorquens* for biosynthesis of PHAs with higher 3HV and 3HHx composition focusing on the EMC pathway involved in C_1_ assimilation. The introduction of ethylmalonyl-CoA decarboxylase, catalyzing a backward reaction in the EMC pathway, aiming to increase intracellular propionyl/butyryl-CoA precursors did not affect PHA composition. Reverse β-oxidation pathway and subsequent (*R*)-specific hydration of 2-enoyl-CoA were then enhanced by heterologous expression of four genes derived from *Ralstonia eutropha* for the conversion of propionyl/butyryl-CoAs to the corresponding (*R*)-3-hydroxyacyl-CoA monomers. The resulting strains produced PHAs with higher 3HV and 3HHx compositions, while the methylotrophic growth was severely impaired. This growth impairment was interestingly restored by the addition of La^3+^ without a negative impact on PHA biosynthesis, suggesting the activation of the EMC pathway by La^3+^. The engineered *M. extorquens* synthesized PHA terpolymer composed of 5.4 mol% 3HV and 0.9% of 3HHx with 41% content from methanol as a sole carbon source in the presence of La^3+^.

## 1. Introduction

Plastic pollution in marine environments is now being recognized as a serious global threat [1,2,3]. It is estimated that microplastics formed from plastic waste will stay in natural environments for hundreds of years due to low degradability of petroleum-based polymers. Polyhydroxyalkanoates (PHAs), which are natural polyesters accumulated within various kinds of microbial cells as a carbon and energy storage, are eco-friendly alternatives to the usual plastics because they show biodegradable properties not only in soil and compost but also in fresh water and sea water. Meanwhile, methanol has received increasing attention as a promising feedstock for the bioindustry based on its sustainability and handleability. Renewable methanol produced from sustainable biomass or CO_2_ and green hydrogen should offer future development of a methanol-based bioeconomy [4]. Hence, PHA production from methanol feedstock has the potential to establish a sustainable plastic industry.

Methylotrophs, capable of growing on reduced C_1_ compounds such as methanol and methylamine as a sole carbon and energy source, are powerful platforms for the establishment of the methanol-based bioeconomy. *Methylorubrum extorquens* (formerly *Methylobacterium extorquens*) AM1 is a model methylotroph for understanding methylotrophy and producing value-added products from methanol. The first step of methanol metabolism in this methylotrophic bacterium is oxidation to formaldehyde catalyzed by methanol dehydrogenase (MDH). *M. extorquens* possesses two distinct types of pyrroloquinoline quinone (PQQ)-dependent MDH, which are Ca^2+^-dependent MxaF and lanthanide (Ln)-dependent XoxF1 and XoxF2. Formaldehyde is then oxidized to formate, which is the major branching node between the catabolic oxidation and anabolic metabolisms. Formate is further oxidized to CO_2_ by formate dehydrogenase, or bound to tetrahydrofolate (H_4_F) to form methylene H_4_F, in which methanol-derived carbon is used to convert glycine to serine in the serine cycle for carbon assimilation [5]. *M. extorquens* AM1 lacking isocitrate lyase utilizes the ethylmalonyl-CoA (EMC) pathway for the regeneration of glyoxylate, a precursor of glycine, to maintain the serine cycle.

*M. extorquens* has been studied well as the producer of single cell proteins as well as several bulk and fine chemicals from methanol [6]. One of the bulk chemicals produced by this bacterium is PHA. The first two steps of the EMC pathway, condensation of two molecules of acetyl-CoA by β-ketothiolase (PhaA) and reduction of acetoacetyl-CoA to (*R*)-3-hydroxybutyryl-CoA (3HB-CoA) by NADPH-dependent acetyl-CoA reductase (PhaB), are shared with the PHA synthesis pathway. The resulting (*R*)-3HB-CoA was dehydrated to crotonyl-CoA for further conversion through the EMC pathway, or polymerized to poly((*R*)-3-hydroxybutyrate) (P(3HB)) by PHA synthase (PhaC). P(3HB) is the most abundant PHA produced by a wide variety of microorganisms in nature, but this homopolyester is unsuitable for applications because of its stiff and brittle properties due to the high crystallinity. It is well known that copolymerization of the 3HB unit with other monomer units such as C_5_-unit (*R*)-3-hydroxyvalerate (3HV), C_6_-unit (*R*)-3-hydroxyhexanoate (3HHx), 4-hyroxybutyrate, and so on, often leads to alternative physical and mechanical properties. Although it had been thought that only P(3HB) was accumulated during methylotrophic growth of *M. extorquens* AM1, we previously revealed that this bacterium is able to synthesize PHA copolymer containing 3HV from methanol under cobalt-deficient conditions [7]. Furthermore, the recombinant strain named AM1C_Ac_, in which original PHA synthase gene (*phaC_Me_*) was replaced by *phaC_Ac_* encoding the homolog with broad substrate specificity from *Aeromonas caviae*, accumulated PHA terpolymer composed of 3HB, 3HV, and additional 3HHx, whereas the 3HV and 3HHx compositions in this terpolymer P(3HB-*co*-3HV-*co*-3HHx) were low (0.71 and 0.28 mol%, respectively) [7].

Recently, an artificial pathway for biosynthesis of P(3HB-*co*-3HHx) from sugars has been constructed in the representative PHA producer *Ralstonia eutropha* (*Cupriavidus necator*) [8,9] In this pathway, the 3HHx monomer was synthesized through the condensation of butyryl-CoA with acetyl-CoA, where butyryl-CoA is provided from crotonyl-CoA by two ways. One is direct formation by reductase activity of crotonyl-CoA carboxylase/reductase (Ccr) derived from *M. extorquens* and another is two step reactions proceeded via reductive carboxylation of crotonyl-CoA and decarboxylation of the resulting ethylmalonyl-CoA catalyzed by the bifunctional Ccr and ethylmalonyl-CoA decarboxylase (Emd) derived from mouse, respectively. As Ccr is one of the key enzymes in the EMC pathway, butyryl-CoA is potentially synthesized in *M. extorquens* under the methylotrophic conditions. Indeed, a small amount of butyryl-CoA was detected in the cell extracts of *M. extorquens* AM1 grown on ethylamine [10,11] However, the reductase activity of Ccr from the strain AM1 was estimated to be much lower than the reductive carboxylase activity [8] as seen for Ccr from *Rhodobacter sphaeroides* [12,13].

In this study, we engineered *M. extorquens* AM1 to increase the supply of 3HV and 3HHx monomer units focusing on the presence of the precursors, propionyl-CoA and butyryl-CoA, in the EMC pathway. The introduction of Emd in combination with the enhancement of reverse β-oxidation (RBO) pathway achieved biosynthesis of PHA with both higher content and compositions of 3HV and 3HHx, whereas the modification in the EMC pathway was accompanied with severe impairment in methylotrophic growth. Interestingly, the impaired methylotrophic growth was restored by the addition of La^3+^ with maintaining the higher PHA content and C_5_/C_6_-monomer composition. This study proposes the potential of lanthanide for the application of the EMC pathway for the production of value-added products from methanol.

## 2. Materials and Methods

### 2.1. Bacterial Strains and Culture Condition

Bacterial strains used in this study are listed in Table 1. *M. extorquens* strains were cultivated at 30 °C in a hypho minimal medium [14] within a 500 mL flask with reciprocal shaking (115 strokes/min). Trace element solution containing EDTA (12.7 g of Na_2_EDTA 2H_2_O, 4.4 g of ZnSO_4_ 7H_2_O, 1.47 g of CaCl_2_ 2H_2_O, 1.01 g of MnCl_2_ 4H_2_O, 0.998 g of FeSO_4_ 7H_2_O, 0.22 g of (NH_4_)_6_Mo_7_O_24_ 4H_2_O, 0.314 g of CuSO_4_ 5H_2_O, and 0.322 g of CoCl_2_ 6H_2_O in 1 L deionized water) was added into the medium (1 mL/1 L medium) except for some cases investigating the effects of metal ions. The standard cultivation of *M. extorquens* on methanol was carried out as described in the previous report [7]. Briefly, the cells pre-cultivated in a 50 mL hypho minimal medium containing 20 mM succinate for 2 days were inoculated into the 100 mL hypho minimal medium containing 125 mM methanol at the initial OD_600_ was 0.1. *E. coli* strains were grown at 37 °C using a Lysogeny broth (LB) medium for general gene manipulation. Kanamycin (100 mg/L) or ampicillin (50 mg/L) was added to the medium for the strains harboring the vector. The cell growth was monitored with optical density (OD) at 600 nm using a JASCO V-550 spectrophotometer (Jasco, Tokyo, Japan).

### 2.2. Construction of Host Strains

General genetic manipulations were performed according to the standard procedures. Primers used in this study are listed in the Supplementary Table S1. 

*M. extorquens* strains AM1C_NSDG_ and AM1C_NSDG__emd were constructed by homologous recombination with pK18mobsacB [15]-based vectors. The plasmid vectors for homologous recombination were constructed as follows. A DNA fragment containing *phaC* with its flanking regions (approximately 1-kbp each) was amplified from *M. extorquens* genomic DNA using a primer set phaC_Me__up1000/phaC_Me__down1000. The amplified fragment was digested by EcoRI and inserted into pK18mobsacB at the corresponding site. Inverse PCR was carried out using the resulting plasmid as a template and a primer set InvphaC_N/InvphaC-C to amplify the flanking regions of *phaC* along with pK18mobsacB backbone. The amplified fragment was ligated with a DNA fragment of *phaC_NSDG_* amplified using pTA2_NSDG [17] and phaC_Ac__N/phaC_Ac__C as a template and a primer set, respectively. The resulting plasmid was designated as pK18_phaC_NSDG_. 

An approximately 2-kbp fragment containing the upstream and downstream regions (approximately 1-kbp each) flanking to the stop codon of *ccr* was amplified from *M. extorquens* genomic DNA using a primer set ccr_Me__C-up1000/ccr_Me__C-down1000. A 5′-phosphorylated PCR product was inserted into pK18mobsacB at the SmaI site. The resulting plasmid was used for inverse PCR as a template with a primer set ccr_Me__C-Inv1/ccr_Me__C-Inv2. The amplified fragment was digested by BamHI, and then ligated with an *emd_Mm_* fragment excised by digestion with BamHI and EcoRV from pTAKN-2-emdMm harboring a synthetic gene encoding mouse ethylmalonyl-CoA decarboxylase ECHDC1 with codons optimized for *R. eutropha* [8]. The resulting plasmid was designated as pK18_emd. 

pK18_phaC_NSDG_ was introduced into *M. extorquens* AM1 by electroporation [18] using a Gene Pulser Xcell^TM^ Electroporation System (Bio-Rad), and the transformant AM1C_NSDG_ was obtained by a pop-in/pop-out recombination as described previously [7]. AM1C_NSDG__emd was obtained by introducing pK18_emd into AM1C_NSDG_ in the same way.

### 2.3. Construction of Expression Plasmids

Several expression vectors for *M. extorquens* strains were constructed using pCM80Km harboring a strong promoter *P_mxaF_* [7,16]. An *emd_Mm_* fragment was amplified using pTAKN-2-emdMm and emd_N/emd_C as a template and a primer set, respectively. The amplified fragment was digested by HindIII and XbaI, followed by ligation into pCM80Km at the corresponding site to obtain pCM80Km_emd. 

A *bktB_Re_* fragment was amplified from *R. eutropha* genomic DNA using a primer set bktB_N/bktB_C, and then digested by BamHI and EcoRI. The digested fragment was inserted into pCM80Km_emd at the corresponding site to obtain pCM80Km_emdbktB.

A tandem of *had_Re_* (H16_A0602)-*crt2_Re_* (H16_A3307) genes was prepared by fusion PCR. The *had_Re_* and *crt2_Re_* fragments were individually amplified from *R. eutropha* genomic DNA using A0602-F/A0602-R-Fus and A3307-F-Fus/A3307-R as the primer sets, respectively. The resulting fragments and a primer set A0602-F/A3307-R were used for fusion PCR, and the fused fragment was cloned into pUC118 at the HincII site. The *had*-*crt2_Re_* fragment amplified from the pUC-hadcrt2 with A0602A3307up/A0602A3307down primers and a *phaJ4a_Re_* fragment amplified from pBPP-J4a [19] with phaJup/phaJdown primers were digested by EcoRI and ligated to each other. The ligation mixture was used as a template for amplification of a *had*-*crt2*-*phaJ4a_Re_* fragment, and the resulting fragment was digested by XbaI and BamHI, followed by insertion into pCM80Km_emdbktB at the corresponding sites to obtain pCM80Km-ehcjb. pCM80Km-hcjb was prepared by excision of the *emd_Mm_* region by digestion with XbaI and HindIII and subsequent blunting and self-ligation.

pCM80PphaA-hcjb was constructed by replacing *P_mxaF_* with a promoter region of *phaA* (*P_phaA_*) in pCM80Km-hcjb. The *P_phaA_* region was amplified using genomic DNA of *M. extorquens* AM1 and PphaA-FwNheI/PphaA-Rv as a template and primer pair, respectively. Inverse PCR was carried out to obtain a linearized pCM80Km-hcjb lacking *P_mxaF_* with primers pCM80-Inv5/A0602-F. These PCR products were digested by NheI and ligated to each other. 

### 2.4. PHA Analyses and Measurement of Methanol Concentration

The cells grown to stationary phase were harvested by centrifugation at 8000× *g* for 10 min at 4°C and washed with deionized water, followed by lyophilization. The dried cells were then subjected to methanolysis in the presence of 15% (*v*/*v*) sulfuric acid in methanol as described previously [20,21]. The reaction mixture was analyzed by gas chromatography using a GC-2014 (Shimadzu, Kyoto, Japan) equipped with an InertCap 1 capillary column (30 m by 0.23 mm; GL Sciences, Tokyo, Japan) and a flame ionization detector. The cellular content and composition were calculated using coefficient obtained by measuring standard PHAs under the same condition.

One mL portion of the culture broth was centrifuged at 17,700× *g*, 1 min at 4 °C, and the supernatants were subjected for determination of methanol concentration using GC-2014 equipped with the same column and detector. 

### 2.5. Methanol Dehydrogenase Assay

The *M. extorquens* strains were cultivated for 48 h after inoculation with an initial OD_600_ 0.1 in 100 mL of hypho medium containing 0.5% methanol with or without 30 mM of LaCl_3_. The cells were harvested by centrifugation at 8000× *g* for 10 min at 4 °C, and then washed with 50 mM Tris-HCl buffer (pH 7.5). The cells resuspended within 20 mM Tris-HCl buffer (pH 7.5) containing 5 mM MgCl_2_ and 1 mM DTT were disrupted by a high-pressure cell disruptor (one-shot model) (Constant Systems, Northants, UK) at 20,000 psi. The cell debris was removed by centrifugation at 8000× *g* for 30 min at 4 °C and the supernatants were used as crude extracts for enzyme assay. Protein concentration was determined by the Bradford method with bovine serum albumin as the standard.

Methanol dehydrogenase (MDH) activity was measured based on the method by Day and Anthony [22] with slight modifications. Briefly, the reaction mixture was composed of 100 mM Tris-HCl buffer (pH 9.0), 15 mM NH_4_Cl, 50 mM 2,6-dichloroindophenol (DCIP), 2 mM phenazine methosulfate (PMS), 1 mM KCN, and 10 mM methanol. The appropriate volume of the crude extract was added into the reaction mixture not containing methanol in a cuvette, and pre-incubated for 5 min at 30 °C for stabilization. The reaction was initiated by the addition of methanol and the substrate-dependent increase in absorbance at 600 nm (ε_600_ = 21 mM^−1^ cm^−1^) was recorded using JASCO V-550 spectrophotometer.

## 3. Results

### 3.1. Introduction of Ethylmalonyl-CoA Decarboxylase into M. extorquens

**Figure 1:**
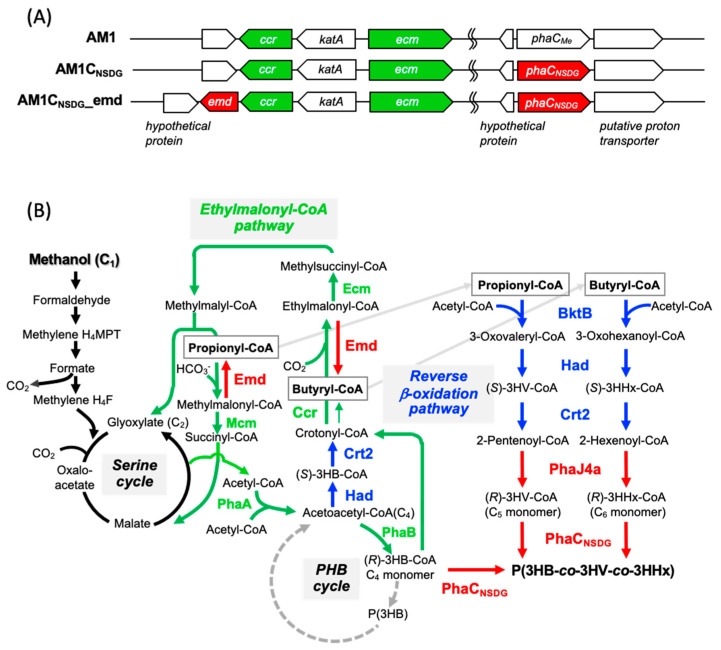
(**A**) Organization of modified genes on chromosome of *M. extorquens* AM1 for PHA biosynthesis. (**B**) Methanol assimilation and P(3HB-*co*-3HV-*co*-3HHx) biosynthesis pathways in *M. extorquens* AM1C_NSDG__emd harboring genes for RBO pathway. 2PG, 2-phosphoglycerate; PEP, phosphoenolpyruvate; PhaA, β-ketothiolase; PhaB, NADPH-acetoacetyl-CoA reductase; Ccr, crotonyl-CoA reductase/carboxylase; Ecm, ethylmalonyl-CoA mutase; Mcm, methylmalonyl-CoA mutase, Emd, ethylmalonyl-CoA decarboxylase; PhaC_NSDG_, N149S/D171G mutant of PHA synthase from *A. caviae*; BktB, short-medium-chain-specific β-ketothiolase; Had, NAD^+^-(*S*)-3-hydroxyacyl-CoA dehydrogenase; Crt2, (*S*)-specific enoyl-CoA hydratase (crotonase); PhaJ4a, medium-chain-specific (*R*)-enoyl-CoA hydratase. BktB, Had, Crt2, and PhaJ4a are derived from *R. eutropha*, and Emd is derived from *Mus musculus* (codon optimized).

We previously demonstrated that *M. extorquens* AM1C_Ac_ harboring PhaC*_Ac_* with broad substrate specificity synthesized P(3HB-*co*-3HV-*co*-3HHx) terpolymers containing small fractions of C_5_ (3HV) and C_6_ (3HHx) units from methanol as a sole carbon source, as described above [7]. In this study, the double mutant (N149S/D171G) of PhaC*_Ac_*, PhaC_NSDG_ showing enhanced ability to incorporate the 3HHx unit [23], was adopted as the polymerizing enzyme in *M. extorquens* (Figure 1A). The resulting strain AM1C_NSDG_ accumulated 31 wt% of the PHA terpolymer per dry cell weight (DCW) with 0.5 mol% 3HV and 0.23 mol% 3HHx compositions after 72 h cultivation on methanol, which were similar to the AM1C_Ac_ strain [7]. Considering the higher 3HHx composition in PHA produced by PhaC_NSDG_-equipped strains of *R. eutropha* and *E. coli* in the previous studies [17,19,23], the little effect of PhaC_NSDG_ on PHA composition strongly implied insufficient provision of the 3HV and 3HHx monomer units from methanol in *M. extorquens*. The strain AM1C_NSDG_ was used as the host strain for further metabolic engineering. 

The C_5_ and C_6_ monomers, 3HV-CoA and 3HHx-CoA respectively, were supposed to be formed from propionyl-CoA and butyryl-CoA in the EMC pathway [7]; the availability of theses precursors for PHA synthesis would affect composition of the accumulated PHA. Emd, firstly identified in mammalian tissues [24], has been reported to show decarboxylation activity towards not only (*S*)-ethylmalonyl-CoA but also (*S*)-methylmalonyl-CoA, where the activity to methylmalonyl-CoA was lower than that to ethylmalonyl-CoA [24]. We therefore introduced Emd into *M. extorquens* AM1C_NSDG_ in anticipation of increasing intracellular concentration of both propionyl-CoA and butyryl-CoA by the backward reaction in the EMC pathway. When *emd_Mm_* was introduced by using an expression vector pCM80Km, the growth of the transformant on methanol was severely impaired. This suggested that the expression of the plasmid-borne *emd_Mm_* under the control of strong methanol dehydrogenase promoter (*P_mxaF_*) was too high to maintain enough turnover of the EMC pathway required for the methylotrophic growth.

It has been reported that specific activity of Ccr in *M. extorquens* was moderately high during the methylotrophic growth [25]. *emd_Mm_* was therefore inserted at downstream of *ccr* on the chromosome of *M. extorquens* AM1C_NSDG_ aiming at the moderate expression (Figure 1A). The resulting strain AM1C_NSDG__emd showed better methylotrophic growth compared to AM1C_NSDG_/pCM80Km-emd, while slight growth retardation was observed on methanol. 

The PHA production by the strains AM1C_NSDG_ and AM1C_NSDG__emd after 72 h cultivation on methanol are summarized in Table 2. Although the PHA content of AM1C_NSDG__emd was 1.5-times higher than that of AM1C_NSDG_, the decrease in DCW of AM1C_NSDG__emd resulted in lower PHA production (0.12 g/L) than that by AM1C_NSDG_ (0.27 g/L). The monomer composition of PHAs produced by these strains were very similar to each other. The cultivation on methanol in the medium supplemented with EDTA-free trace element solution was further examined, because we have previously observed that the methylotrophic growth rate of *M. extorquens* AM1 was increased in the absence of EDTA. This was speculated to be due to higher metabolic flux of the EMC pathway attributed to higher activity of two vitamin B_12_-dependent mutases in the EMC pathway, ethylmalonyl-CoA mutase and methylmalonyl-CoA mutase, under high concentration of Co^2+^ without chelation by EDTA [7,26]. Both DCW and PHA production by the strain AM1C_NSDG__emd were restored to comparable level to those of AM1C_NSDG_ in the absence of EDTA, whereas the 3HV and 3HHx fractions in the accumulated PHA were slightly reduced (Table 2).

### 3.2. Enhancement of the Reverse β-Oxidation (RBO) Pathway

Despite the poor growth on methanol caused by Emd*_Mm_* catalyzing decarboxylation opposite to the usual direction of the EMC pathway, no significant increase in fractions of 3HV and 3HHx was observed, suggesting insufficient function of the native pathway for conversion of propionyl/butyryl-CoAs to the corresponding (*R*)-3-hydroxyacyl (3HA)-CoA monomers. Recently, we enhanced the RBO pathway responsible for the conversion of butyryl-CoA to (*R*)-3HHx-CoAs in *R. eutropha* for biosynthesis of P(3HB-*co*-3HHx) from glucose [9]. In the enhanced RBO pathway, β-ketothiolase (BktB*_Re_*), NAD^+^-dependent (*S*)-3HA-CoA dehydrogenase (Had*_Re_*), and (*S*)-specific enoyl-CoA hydratase (crotonase) (Crt2*_Re_*) potentially formed 2-enoyl-CoAs of C_5_/C_6_ from propionyl/butyryl-CoAs and acetyl-CoA via (*S*)-3HA-CoAs. Then, (*R*)-enoyl-CoA hydratase (PhaJ4a*_Re_*) converted 2-enoyl-CoAs to (*R*)-3HA-CoAs. The four enzymes derived from *R. eutropha*, all of which have been demonstrated to show rather broad substrate specificity accepting the medium-chain-length intermediates [19,27], were applied in *M. extorquens* to promote the supply of the 3HV/3HHx-monomer units (Figure 1B). When an artificial cluster of *had*-*crt2*-*phaJ4a-bktB_Re_* flanked to the strong promoter *P_mxaF_* was introduced into *M. extorquens* AM1C_NSDG__emd by using the plasmid vector pCM80Km-hcjb, the resulting transformant showed slower methylotrophic growth than the host strain AM1C_NSDG__emd. Because we concerned that activities of the heterologous enzymes competing with the EMC pathway were too high, another vector was constructed by replacing *P_mxaF_* with a putative promoter region of *phaA* (Mex_1p3700) expecting moderate expression of the genes. However, contrary to expectation, the growth of the strain harboring the resulting vector pCM80PphaA-hcjb on methanol was further impaired in comparison with the strain harboring pCM80Km-hcjb.

### 3.3. PHA Production by the Engineered Strains of M. extorquens

**Figure 2:**
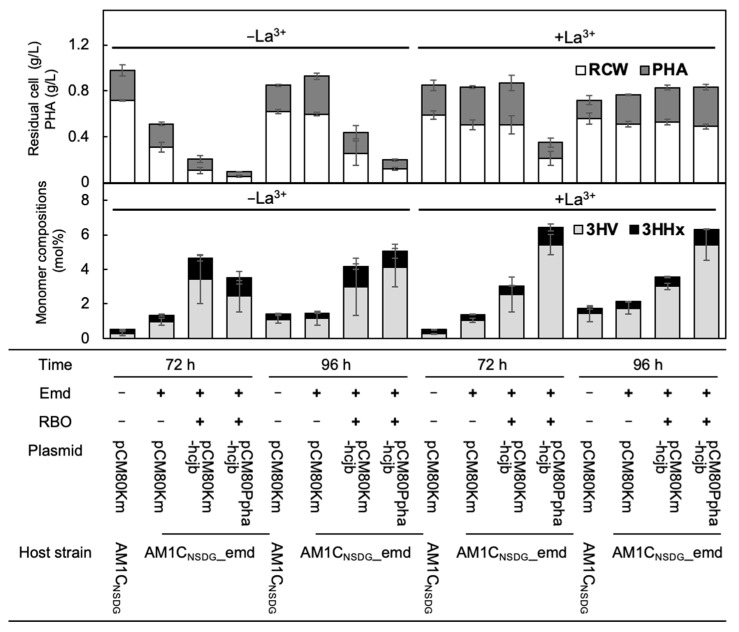
P(3HB-*co*-3HV-*co*-3HHx) biosynthesis from methanol by engineered strains of *M. extorquens* in the absence or presence of La^3+^. The cells were grown in 100 mL hypho medium containing 0.5% (*v*/*v*) methanol and trace element solution with EDTA for 72 h or 96 h. *n* = 4 (*n* = 2 for AM1_NSDG_/pCM80Km (72 h)); error bars represent standard deviations.

Although the strain AM1C_NSDG__emd, in which *emd* was introduced onto the chromosome, showed poor growth at 72 h on methanol under the metal-deficient condition (EDTA^+^) (Table 2), the dry cell weight of this strain harboring the empty vector pCM80Km reached comparable level with that of AM1C_NSDG_/pCM80Km after a prolonged 96 h cultivation (Figure 2). Therefore, PHA synthesis by the engineered strains was evaluated after 96 h of cultivation (Figure 2, Table 3). PHA content in AM1C_NSDG__emd/pCM80Km was slightly higher than that in AM1C_NSDG_/pCM80Km, and the PHA composition was not changed by introducing Emd*_Mm_*. The strains with the enhanced RBO pathway, AM1C_NSDG__emd/pCM80Km-hcjb and AM1C_NSDG__emd/pCM80PphaA-hcjb, produced PHAs consisted of 4.1–4.2 mol% 3HV and 0.9–1.1 mol% 3HHx composition, which were higher than those of PHA produced by the control strain AM1C_NSDG__emd/pCM80Km. These RBO-enhanced strains showed PHA contents as high as 40 wt%, whereas the PHA production was lower than that by the control strain due to the lower cell growth (dry cell weight). 

### 3.4. Both Methylotrophic Growth and PHA Production Were Restored by Addition of La^3+^

**Figure 3:**
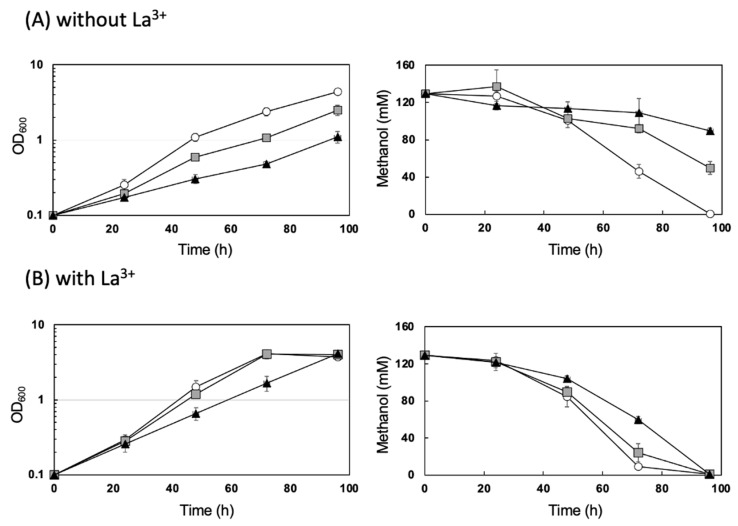
Time courses of growth of and methanol consumption by engineered strains of *M. extorquens*. The cells were grown in 100 mL hypho medium containing 0.5% (*v*/*v*) methanol and trace element solution with EDTA in the absence (**A**) or presence (**B**) of La^3+^. Open circle, AM1C_NSDG__emd/pCM80Km; gray square, AM1C_NSDG__emd/pCM80Km-hcjb; closed triangle, AM1C_NSDG__emd/pCM80PphaA-hcjb. *n* ≥ 3; error bars represent standard deviations.

The enhancement of the RBO pathway in *M. extorquens* resulted in impaired growth on methanol (Figure 3A). The determination of residual methanol concentration in the media indicated that approximately 70% of the initial methanol remained even after 96 h cultivation of AM1C_NSDG__emd/pCM80PphaA-hcjb showing the lowest methylotrophic growth among the strains examined (Figure 3A). AM1C_NSDG__emd/pCM80Km-hcjb using *P_mxaF_* for expression of the heterologous genes showed slow growth and methanol consumption as well (Figure 3A). 

Given the much larger metabolic flux of methanol oxidation than C_1_ assimilation for the conservation of energy and reducing equivalents required for the growth [28], it was assumed that the repression of methanol oxidation may have some relation to the poor methylotrophic growth of the engineered strains. The methanol oxidation is mainly catalyzed by Ca^2+^-dependent MDH (MxaFI) in *M. extorquens* AM1, while recent studies revealed that this methylotroph possesses the second MDH, the lanthanide-dependent XoxF1, and the expression and activity of XoxF1 are induced under the presence of lanthanide [29,30]. We thus investigated the effects of lanthanide on the growth of the engineered strains of *M. extorquens*, and interestingly observed that the poor methylotrophic growth and methanol consumption of the RBO-enhanced strains were both restored by the addition of 30 mM LaCl_3_ into the culture medium (Figure 3B). AM1C_NSDG__emd/pCM80Km and AM1C_NSDG__emd/pCM80Km-hcjb showed maximal dry cell weight (residual cell plus PHA) at 72 h of cultivation in the presence of La^3+^ (Figure 2). Notably, both PHA content and compositions of 3HV and 3HHx were maintained as high as those in the absence of La^3+^ (Figure 2, Table 3). It was initially thought that the reduced activity of MxaFI in the engineered strains might be compensated by the induction of XoxF1 in the presence of La^3+^. This was consistent with slightly higher MDH activity in AM1C_NSDG__emd/pCM80Km-hcjb cultivated with La^3+^ (274 mU/mg protein) than that in the absence of La^3+^ (173 mU/mg protein). However, AM1C_NSDG__emd/pCM80Km control strain showed MDH activity of 197–234 mU/mg protein regardless of the presence or absence of La^3+^. It would be feasible that the change of MDH activity in the RBO-enhanced strain by addition of La^3+^ was not related to the restoration of methylotrophic growth.

## 4. Discussion

The EMC pathway, specific and essential for methylotrophy of *M. extorquens* and the related methylotrophs, is an attractive pathway for the production of value-added compounds from methanol because this pathway contains CoA-thioester intermediates with various structures. We have previously found that propionyl/butyryl-CoA intermediates in the EMC pathway were the potential precursors of P(3HB-*co*-3HV-*co*-3HHx) terpolymer in *M. extorquens* under Co^2+^-deficient condition [7]. The Co^2+^-dependency was thought to be attributed to the activity levels of the two B_12_-dependent mutases (ethylmalonyl-CoA mutase and methylmalonyl-CoA mutase) in the EMC pathway, responsible for the intracellular concentration of propionyl/butyryl-CoAs. In this study, Emd*_Mm_* was introduced into *M. extorquens* to increase the availability of propionyl/butyryl-CoAs for PHA synthesis through converting methylmalonyl/ethymalonyl-CoAs back to the short-chain acyl-CoAs. However, the expression of *emd_Mm_* resulted in growth inhibition on methanol, which was agreed with reduced flux of the EMC pathway weakened by the Emd*_Mm_*-mediated backward reactions. This growth inhibition was restored by using an EDTA-free trace element solution for the cultivation, probably due to compensation by activation of the two mutases under the high Co^2+^ concentration. These results again demonstrated the strong association of the EMC pathway with the methylotrophic growth of *M. extorquens*. Considering the 20-times higher catalytic efficiency (*k*_cat_/*K*_m_) of Emd*_Mm_* toward ethylmalonyl-CoA than that to methylmalonyl-CoA, ethylmalonyl-CoA mutase may play a significant role in the methylotrophic growth.

Unfortunately, the introduction of Emd*_Mm_* into *M. extorquens* AM1C_NSDG_ gave almost no effect on PHA composition despite the function of PhaC_NSDG_ and potentially larger pool of propionyl/butyryl-CoAs in the weakened EMC pathway. As it was supposed that the native pathway for conversion of propionyl/butyryl-CoAs to (*R*)-3HA-CoAs did not work well in *M. extorquens*, further modification was conducted to enhance RBO pathway. In the last decade, RBO pathway has been applied for elongation of acetyl-CoA to higher alcohols, aldehydes, monocarboxylic acids, and so on [31]. Here, four enzymes involved in RBO (BktB, Had, Crt2) and subsequent formation of (*R*)-3HA-CoAs from 2-enoyl-CoAs (PhaJ4a), which are all derived from and applied in *R. eutropha* [9], were introduced into *M. extorquens*. The resulting strain AM1C_NSDG_/pCM80Km-hcjb produced the PHA terpolymer with about a 3-times higher composition of 3HV and 3HHx units from methanol when compared with that produced by AM1C_NSDG_/pCM80Km. In general, copolymerization of 3HB and other hydroxyalkanoate unit tends to improve the hard and brittle properties of P(3HB) homopolymer. In the case of P(3HB-*co*-3HHx), increase in the 3HHx fraction from 0 to 10 % elevated elongation to break from 5 to 400% accompanied with a decrease in crystallinity [32]. Another study reported that only 1.5 mol% of 3HHx unit in the copolymer chain markedly decreased the melting temperature from 175 °C to 150 °C [33]. Such effect of the copolymerization is not very significant when the second unit is 3HV, because 3HV is co-crystallized with 3HB, thus elongation to break of P(3HB-*co*-20 mol% 3HV) was only 50% [32]. Interestingly, the co-crystallization of 3HB and 3HV seemed to be prevented by further co-polymerization with 3HHx [34]. The terpolymers produced by the engineered *M. extorquens* strains from methanol is expected to exhibit higher flexibility than P(3HB) homopolymer, despite the low composition of ~4 mol% 3HV and ~1 mol% 3HHx. Further engineering of the strains for the synthesis of PHAs with higher C_5_/C_6_ composition and characterization of the polymer will be an important issue to be investigated.

**Figure 4:**
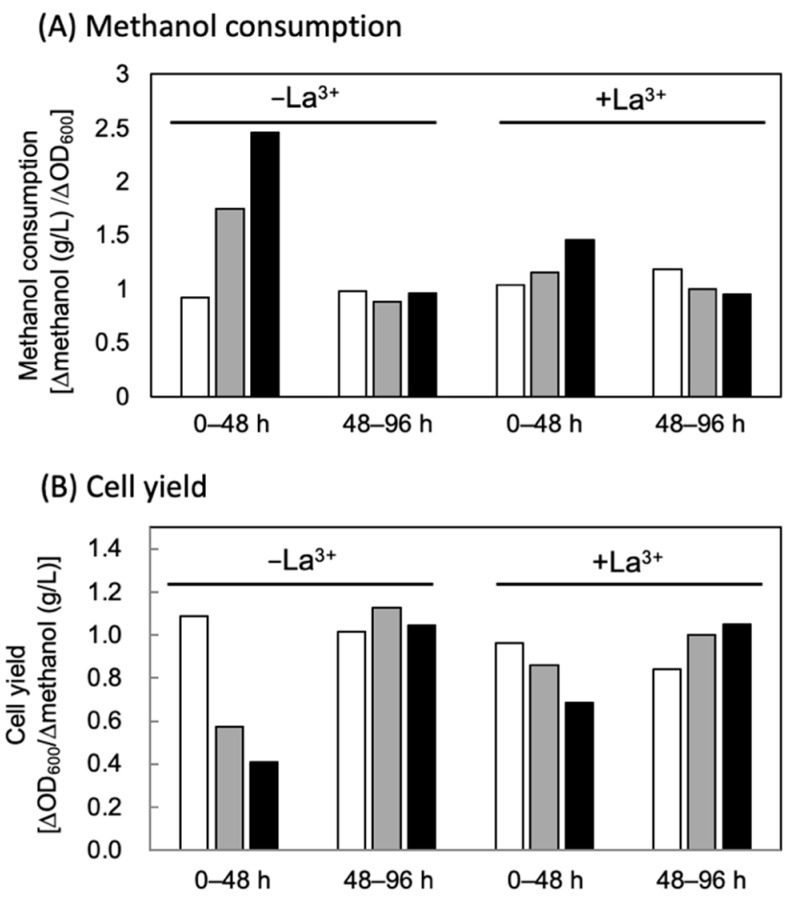
Cellular methanol consumption (**A**) and cell yield to methanol (**B**) of engineered strains of *M. extorquens* during initial-mid (0–48 h) and mid-late (48–96 h) phases. The cells were grown in 100 mL hypho medium containing 0.5% (*v*/*v*) methanol and trace element solution with EDTA. Open bars, AM1C_NSDG__emd/pCM80Km; Gray bars, AM1C_NSDG__emd/pCM80Km-hcjb; Closed bars, AM1C_NSDG__emd/pCM80PphaA-hcjb.

The high 3HV and 3HHx monomer composition in PHA synthesized by the RBO-enhanced strains was consistent with channeling of more propionyl/butyryl-CoA intermediates from the EMC pathway to PHA synthesis. Meanwhile, the enhancement of the RBO pathway accompanied severe impairment in the methylotrophic growth under the Co^2+^ -deficient condition, and moreover, the reduction of methanol consumption was observed. These trends were common between the strains using different promoter (*P_mxaF_* or *P_phaA_*) for expression of the RBO pathway genes. The reduction of the overall methanol consumption by the RBO-enhanced strains was thought to be a result of the lower cell concentration in the culture medium. Here we noticed that the cellular methanol consumption by the RBO-enhanced strains were higher during the early growth phase (0–48 h) when compared to that by AM1C_NSDG__emd harboring the pCM80Km empty vector (Figure 4A). Namely, the cell yields of the RBO-enhanced strains to methanol were decreased in this term (Figure 4B). Metabolomics analysis of *M. extorquens* revealed that only 16% of the consumed methanol was assimilated via H_4_F-dependent C_1_ transfer and the remaining 86% was completely oxidized to CO_2_ for energy conservation [28]. Considering the present observation that the RBO-enhanced strain showed no significant decrease in MDH activity, it implies that the reduction of the EMC pathway flux caused by the enhancement of RBO pathway did not significantly affect the methanol catabolism. Impairment of cell growth by the reduction of the EMC pathway lowered cell yield but only at the early phase. 

The above results again indicated tradeoff between cell growth and metabolic flux channeled from the EMC pathway, which is a serious problem for the use of the acyl-CoA intermediates for the production of value-added compounds. Recently, regulation of cellular functions by lanthanides (Ln switch) [35] has been recognized to be important for methylotrophy of several methylotrophs and methanotrophs. In this study, we found a possibility that the Ln switch would be useful to overcome the above tradeoff, as the poor cell yields of the engineered *M. extorquens* strains to methanol was restored by the addition of La^3+^. It should be noted that the higher 3HV and 3HHx compositions in PHA synthesized on methanol were maintained during the growth restored by La^3+^ (Figure 2), unlike the growth restoration by the high concentration of Co^2+^ accompanied with a decrease in the 3HV/3HHx composition of PHA (Table 2). It was thought that carbon flux of the EMC pathway was increased by the addition of La^3+^. However, previous transcriptomic analyses of *Methylorubrum* species demonstrated that expression levels of genes in C_1_-assimilation pathways including H_4_F-dependent C_1_ transfer, the serine cycle, and the EMC pathway, were not up-regulated by lanthanide, in contrast to down-regulation of *mxa* and up-regulation of *xox* clusters [36,37]. This rose a possibility for some regulation of C_1_-assimilation pathways by La^3+^ other than transcriptional induction, such as protein-level activation of some key enzymes in the assimilation pathways. Alternatively, transcriptional behavior to La^3+^ may be altered when the EMC pathway was modified. Further efforts should be made to understand the detailed mechanism and role of Ln switch in *M. extorquens*. We here achieved flask-scale methylotrophic growth of *M. extorquens* up to 0.8 g/L dry cells containing 41.5 wt% P(3HB-*co*-5.4 mol% 3HV-*co*-0.9 mol% 3HHx) under La^3+^- adding condition. A previous studies have demonstrated high-cell-density cultivation of *M. extorquens* on methanol (114–132 g-dry cells/L) by fed-batch strategy [38,39]. The fed-batch cultivation of the engineered strains in the presence of La^3+^ will allow the efficient production of PHA terpolymers from methanol. Taken together, the results of this study suggested that the response of methylotrophic bacteria to lanthanides would be beneficial for the application of the EMC pathway for bioproduction.

## Figures and Tables

**Table 1:** Strains and plasmids used in this study.

Strains or Plasmids	Relevant Characteristics	Source or Reference
***Escherichia coli***		
DH5α	*deoR*, *endA1*, *gyrA96*, *hsdR17* (r_K_^−^ m_K_^+^), *recA1*, *relA1*, *supE44*, *thi-l*, ∆(*lacZYA-argFV169*), φ80*lacZ*∆M15, F^−^, λ^−^	Clontech (Palo Alto, CA)
***Methylorubrum extorquens***		
AM1	Wild type	
AM1C_NSDG_	AM1 derivative; *phaC_Me_*::*phaC*_NSDG_	This study
AM1C_NSDG__emd	AM1C_NSDG_ derivative; *emd* downstream of *ccr*	This study
**Plasmids**		
pK18mobsacB	pMB1 ori, RP4 *mob*, modified *sacB*, *lacZa*, Kan^r^	[15]
pK18_ phaC_NSDG_	pK18mobsacB carrying phaC_NSDG_	This study
pTAKN-2-emdMm	pTAKN-2 cloning vector carrying *emd_Mm_*	[8]
pK18_emd	pK18mobsacB carrying *emd_Mm_*	This study
pCM80Km	pCM80 derivative; Tc^r^::Kan^r^	[7,16]
pCM80Km_emd	pCM80Km carrying *emd_Mm_*	This study
pCM80Km_emdbktB	pCM80Km carrying *emd_Mm_*-*bktB_Re_*	This study
pUC118	Amp^r^ general cloning vector	Takara Bio (Ohtsu, Japan)
pUC-hadcrt2	pUC118 carrying *had_Re_*-*crt2_Re_*	This study
pCM80Km-ehcjb	pCM80Km carrying *emd_Mm_*-*had_Re_*-*crt2_Re_*-*phaJ4a_Re_*-*bktB_Re_*	This study
pCM80Km-hcjb	pCM80Km carrying *had_Re_*-*crt2_Re_*-*phaJ4a_Re_*-*bktB_Re_*	This study
pCM80PphaA-hcjb	pCM80Km-hcjb derivative; *P_mxaF_*::*P_phaA_*	This study

**Table 2:** P(3HB-*co*-3HV-*co*-3HHx) biosynthesis by *M. extorquens emd*-introducing strains from methanol.

Strain	EDTA	Dry Cell Weight (g/L)	PHA Content (wt%)	PHA (g/L)	Residual Cell Weight (g/L)	Monomer Composition (mol%)		
						3HB	3HV	3HHx
AM1C_NSDG_	+	0.96 ± 0.11	28.0 ± 2.6	0.27 ± 0.02	0.69 ± 0.10	99.3 ± 0.1	0.45 ± 0.09	0.23 ± 0.02
AM1C_NSDG__emd		0.28 ± 0.06	42.5 ± 8.1	0.11 ± 0.01	0.16 ± 0.06	99.3 ± 0.2	0.39 ± 0.20	0.33 ± 0.01
AM1C_NSDG_	−	1.11 ± 0.07	33.1 ± 1.9	0.37 ± 0.01	0.74 ± 0.07	99.6 ± 0.0	0.18 ± 0.01	0.18 ± 0.01
AM1C_NSDG__emd		1.08 ± 0.03	23.1 ± 1.0	0.25 ± 0.01	0.83 ± 0.02	96.6 ± 0.0	0.20 ± 0.01	0.17 ± 0.02

**Table 3:** Effect of lanthanide on P(3HB-*co*-3HV-*co*-3HHx) biosynthesis by *M. extorquens* recombinant strains from methanol.

Strain	Plasmid	La	Dry cell Weight (g/L)	PHA Content (wt%)	PHA (g/L)	Residual Cell Weight (g/L)	Monomer Composition (mol%)		
							3HB	3HV	3HHx
AM1C_NSDG_	pCM80Km	−	0.85 ± 0.02	27.1 ± 1.2	0.23 ± 0.01	0.62 ± 0.02	98.6 ± 0.3	1.09 ± 0.22	0.31 ± 0.04
	pCM80Km-emd		0.07 ± 0.01	27.4 ± 1.7	0.02 ± 0.00	0.05 ± 0.01	98.1 ± 0.3	0.97 ± 0.21	0.89 ± 0.11
AM1C_NSDG__emd	pCM80Km		0.93 ± 0.04	35.5 ± 1.8	0.33 ± 0.03	0.59 ± 0.02	98.5 ± 0.4	1.15 ± 0.40	0.32 ± 0.03
	pCM80Km-hcjb		0.44 ± 0.16	42.3 ± 2.8	0.18 ± 0.05	0.26 ± 0.10	95.8 ± 1.7	3.00 ± 1.67	1.17 ± 0.15
	pCM80PphaA-hcjb		0.20 ± 0.02	39.7 ± 2.1	0.08 ± 0.01	0.12 ± 0.01	95.0 ± 1.4	4.11 ± 1.12	0.94 ± 0.40
AM1C_NSDG_	pCM80Km	+	0.72 ± 0.09	22.2 ± 2.5	0.16 ± 0.04	0.56 ± 0.05	98.3 ±0.5	1.43 ± 0.48	0.32 ± 0.05
	pCM80Km-emd		0.61 ± 0.06	27.2 ± 0.8	0.17 ± 0.02	0.44 ± 0.04	97.9 ± 0.1	1.55 ± 0.04	0.55 ± 0.04
AM1C_NSDG__emd	pCM80Km		0.77 ± 0.02	33.6 ± 1.6	0.26 ± 0.01	0.51 ± 0.02	97.9 ± 0.3	1.75 ± 0.34	0.37 ± 0.02
	pCM80Km-hcjb		0.83 ± 0.02	36.2 ± 2.3	0.30 ± 0.02	0.53 ± 0.02	96.4 ± 0.2	3.02 ± 0.17	0.55 ± 0.04
	pCM80PphaA-hcjb		0.83 ± 0.01	41.3 ± 2.6	0.34 ± 0.02	0.49 ± 0.02	93.7 ± 1.0	5.42 ± 0.89	0.90 ± 0.12

## Data Availability

The data presented in this study are available in the article or Supplementary Materials.

## Supplementary Materials

The following are available online at https://www.mdpi.com/2076-2607/10/1/184/s1, Table S1: The sequences of primers used in this study.
