# Correction: Orita et al. Biosynthesis of Polyhydroxyalkanoate Terpolymer from Methanol via the Reverse β-Oxidation Pathway in the Presence of Lanthanide. *Microorganisms* 2022, *10*, 184

**DOI:** 10.3390/microorganisms10030529

**Published:** 2022-02-28

**Authors:** Izumi Orita, Gento Unno, Risa Kato, Toshiaki Fukui

**Affiliations:** School of Life Science and Technology, Tokyo Institute of Technology, 4259 Nagatsuta, Midori-ku, Yokohama 226-8501, Japan; orita.i.aa@m.titech.ac.jp (I.O.); gento910@gmail.com (G.U.); kato.r.ac@m.titech.ac.jp (R.K.)

The authors wish to make the following corrections to this paper [1]:

The authors note that Figure 4B appeared incorrectly in the published version of the paper. The *y*-axis is not labeled in the published figure.

The correct version of Figure 4 is as follows:

The authors would like to apologize for any inconvenience caused to readers by these changes.

**Figure 4:**
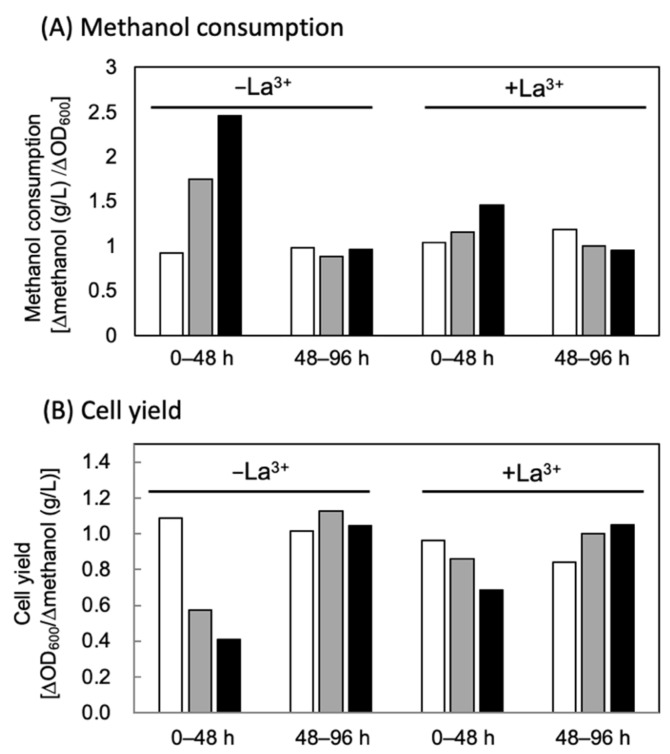
Cellular methanol consumption (**A**) and cell yield to methanol (**B**) of engineered strains of *M. extorquens* during initial-mid (0–48 h) and mid-late (48–96 h) phases. The cells were grown in 100 mL hypho medium containing 0.5% (*v*/*v*) methanol and trace element solution with EDTA. Open bars, AM1C_NSDG__emd/pCM80Km; Gray bars, AM1C_NSDG__emd/pCM80Km-hcjb; Closed bars, AM1C_NSDG__emd/pCM80PphaA-hcjb.
