# Supplementary material for: Biosynthesis of Polyhydroxyalkanoate Terpolymer from Methanol via the Reverse β-Oxidation Pathway in the Presence of Lanthanide
Source: Microorganisms. 2022 Jan 15;10(1):184. doi: 10.3390/microorganisms10010184 (PMC8780949; doi:10.3390/microorganisms10010184)
Supplement: Supplementary file 1 [file microorganisms-10-00184-s001.zip › microorganisms-1462903-supplementary.pdf]

Table S1. List of primers used in the study.

**Primer**	**Sequence (5′-3′)**
Construction of pK18_ phaC$_{\mathrm{NSDG}}$	
phaC$_{\mathrm{Me}}$_up1000	cggaattcGAAGCCGAAGGCGTGGATCGG
phaC$_{\mathrm{Me}}$_down1000	cggaattcGACGATGCCGGGATCGAACAGCATG
InvphaC_N	GCGACACGTCCTCCCAAAGGTCCG
InvphaC-C	GGGCATGAAGGTGTGAGGGATCG
phaC$_{\mathrm{Ac}}$_N	ATGAGCCAACCATCTTATGGCCCGC
phaC$_{\mathrm{Ac}}$_C	TCATGCGGCGTCCTCCTCTGTTGGG
Construction of pK18_emd	
ccr$_{\mathrm{Me}}$_C-up1000	ACGTGCACAAGGGCGAGTACCACATCGC
ccr$_{\mathrm{Me}}$_C-down1000	GACCGGCGTCGCCTCTCGCATTCG
ccr$_{\mathrm{Me}}$_C-Inv1	CGCGGATCCTCACATCGCCTTGAGCGGGCCG
ccr$_{\mathrm{Me}}$_C-Inv2	GCCAGTCCACGTGACACGAAGAAG
Construction of pCM80Km_emd	
emd_N	ACATGCATGCAAGCTTAGGGAGTTCCATATGGCCAAGTGCCTGCTGACGT
emd_C	GGAATTCCGGATCCTCTAGACTACTTGGTGTGCTTGCCCTTCTTCGCGATG
Construction of pCM80Km_emdbktB	
bktB_N	CGGGATCCAGGGAGCAAAGTCATGACGCGTGAAGTGGTAGTGGTA
bktB_C	GGGAATTCTCAGATACGCTCGAAGATGGCGGCAATGCCC
Construction of pCM80Km-ehcjb and pCM80Km-hcjb	
A0602-F	ATGCAAATCCAAGGCAACGTATTCA
A0602-R-Fus	ATGTATTTGCCTTTACTTGGGCTGCATCCGGA
A3307-F-Fus	CCAAGTAAAGGCAAATACATAGGAGAAGACA
A3307-R	GCCTTAGCGATGCTGGAAATT
A0602A3307up	GCTCTAGAAGGGAGATTATCATATGCAAATCCAAGGCAACGTATTCAT
A0602A3307down	GGAATTCAGGGAGCTATCGACATGCGTACCATCGCATCGCTGGA
phaJup	GGAATTCTTAGCGATGCTGGAAATTCGGGCTG
phaJdown	CGGGATCCTCACCCGTAGCGGCGCGTGATCGACT
Construction of pCM80PphaA-hcjb	
PphaA-FwNheI	GTTCTCGCTAGCCGTTGCGACACCTCCCGCTTC
PphaA-Rv	CGTGACGTTCTCCTCATTCGGC
pCM80-Inv5	CCCGACCAAGCGGGAAGCTAGCT
